# Case Report: Bullous Pemphigoid Associated With Morphea and Lichen Sclerosus: Coincidental Diseases or Pathogenetic Association?

**DOI:** 10.3389/fimmu.2022.887279

**Published:** 2022-05-03

**Authors:** Roberto Maglie, Maria Efenesia Baffa, Francesca Montefusco, Carlo Pipitò, Stefano Senatore, Marco Capassoni, Vincenza Maio, Marco Matucci Cerinic, Emiliano Antiga, Serena Guiducci

**Affiliations:** ^1^ Department of Health Sciences, Section of Dermatology, University of Florence, Florence, Italy; ^2^ Department of Experimental and Clinical Medicine, University of Florence, Florence, Italy; ^3^ Department of Geriatric Medicine, Division of Rheumatology and Scleroderma Unit Azienda Ospedaliera Universitaria Careggi (AOUC), Florence, Italy; ^4^ Department of Health Sciences, Division of Pathological Anatomy, University of Florence, Florence, Italy

**Keywords:** bullous pemphigoid, morphea, lichen sclerosus, BP180, autoantigen

## Abstract

Bullous pemphigoid (BP) represents the most common autoimmune bullous disease and is characterized by IgG autoantibodies targeting collagen XVII (BP180). BP has reportedly been occurred in association with other inflammatory skin diseases. Here, we describe the unusual occurrence of BP in a female patient with a concomitant history of generalized morphea (localized scleroderma, LoS) and cutaneous and genital lichen sclerosus (LiS). The occurrence of BP was associated with elevated serum levels of anti-BP180 IgG autoantibodies, which decreased upon clinical remission. Autoimmune bullous diseases and sclerosing dermatitis are immunologically distinct entities, whose association has been rarely described. In this study, we provide a literature review on cases of BP developed in patients with either LoS or LiS. Further, we discussed immunological mechanisms which may have favored the emergence of BP in our patient.

## Introduction

Bullous pemphigoid (BP) is an autoimmune bullous disease that prevalently affects the elderly (1). The pathogenesis of BP is related to IgG autoantibodies targeting collagen XVII, also referred to as BP180, and particularly the non-collagenous domain NC16A. Antibody/antigen binding destabilizes the adhesion function of BP180, induces complement activation and attracts various inflammatory cells, including neutrophil and eosinophil granulocytes, eventually leading to increased expression of inflammatory cytokines and secretion of proteolytic enzymes (2–5). Collectively, these events lead to dermal- epidermal detachment. Antibodies targeting BP230 develop in most BP patients due to intermolecular epitope spreading, but demonstrate pathogenicity in animal models as well as correlation with disease activity in humans (6, 7).

Classic clinical presentation of BP features erythema, urticarial plaques, blisters and erosions; non-bullous variants, including eczematous or prurigo-like forms, have been also described (8). Rare variants include Brusting-Perry pemphigoid (9) and laminin γ1 pemphigoid (10).

The emergence of BP is sometimes precipitated by an external or internal trigger, including drugs (11, 12), vaccines (13), or malignancies (14). Localized forms can also arise on sites of previously damaged skin, e.g. following radiotherapy (15), or surgical procedures (16), and can be followed by generalized spreading (16). Finally, a previous history of an inflammatory skin disease, including psoriasis, atopic dermatitis, and dermatitis herpetiformis, may confer susceptibility to the development of BP (17–19). Here, we discuss a late occurrence of BP in a patient with a long history of morphea (localized scleroderma, LoS) and lichen sclerosus (LiS).

## Case Description

In 2019, a 77-year-old woman attended our clinic due to a 1-year history of recalcitrant and pruritic blisters and erosions affecting the forearms. She had a 25-year history of cutaneous and genital Lis combined with generalized LoS, both confirmed by histopathological examination. Over the past years, she was managed with multiple lines of topical and systemic steroids, UVA1 phototherapy (the last cycle in 2014) and methotrexate. When she was referred to us, she was on methotrexate 15mg once a week and oral prednisone 5 mg per day.

**Figure 1:**
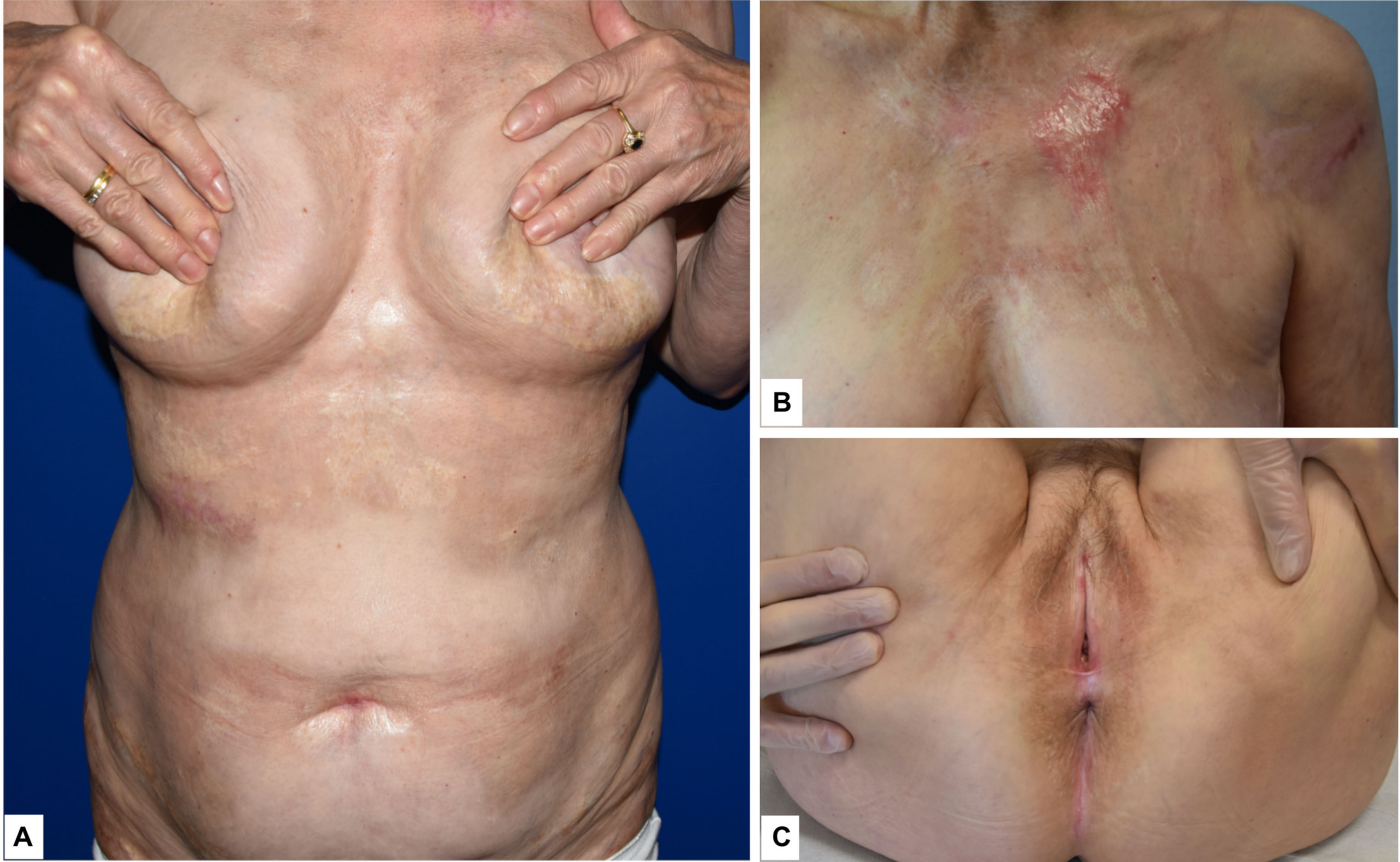
**(A)** Whitish indurated plaques with slight erythematous border consistent with localized scleroderma; **(B)** detail of the patient’s trunk, where a whitish indurated lesion could be observed; **(C)** erythema and scarring around the anogenital area of the patient consistent with lichen sclerosus.

**Figure 2:**
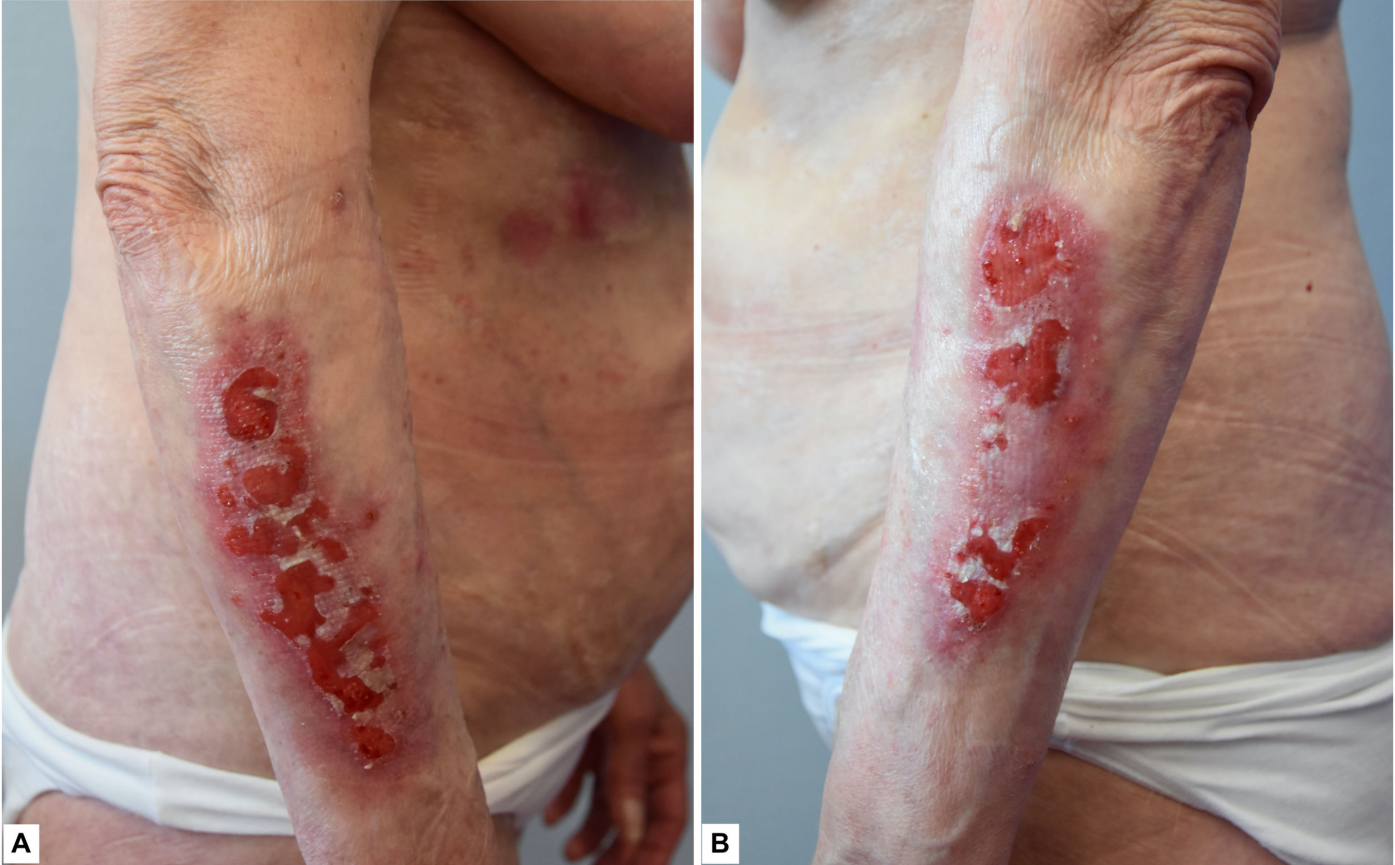
**(A, B)** Erosions superimposed on whitish plaques with atrophic epidermis at the right and left upper limbs.

Physical examination demonstrated multiple whitish indurated plaques distributed at the trunk, upper and lower limbs, as well as at the genitalia, consistent with the patient’s history of LoS and LiS (**Figures 1A–C**). Examination of the forearms demonstrated confluent erosions superimposed on skin areas affected by LoS and LiS lesions (**Figures 2A, B**). There was no evidence of blisters.

Lab tests did not reveal significant abnormalities. Anti-nuclear, anti-histone and anti-single stranded DNA antibodies were negative. Our diagnostic work-up included light microscopy examination and immunopathological studies to detect either tissue-bound or circulating autoantibodies to epidermal-basement membrane zone (BMZ) antigens.

**Figure 3:**
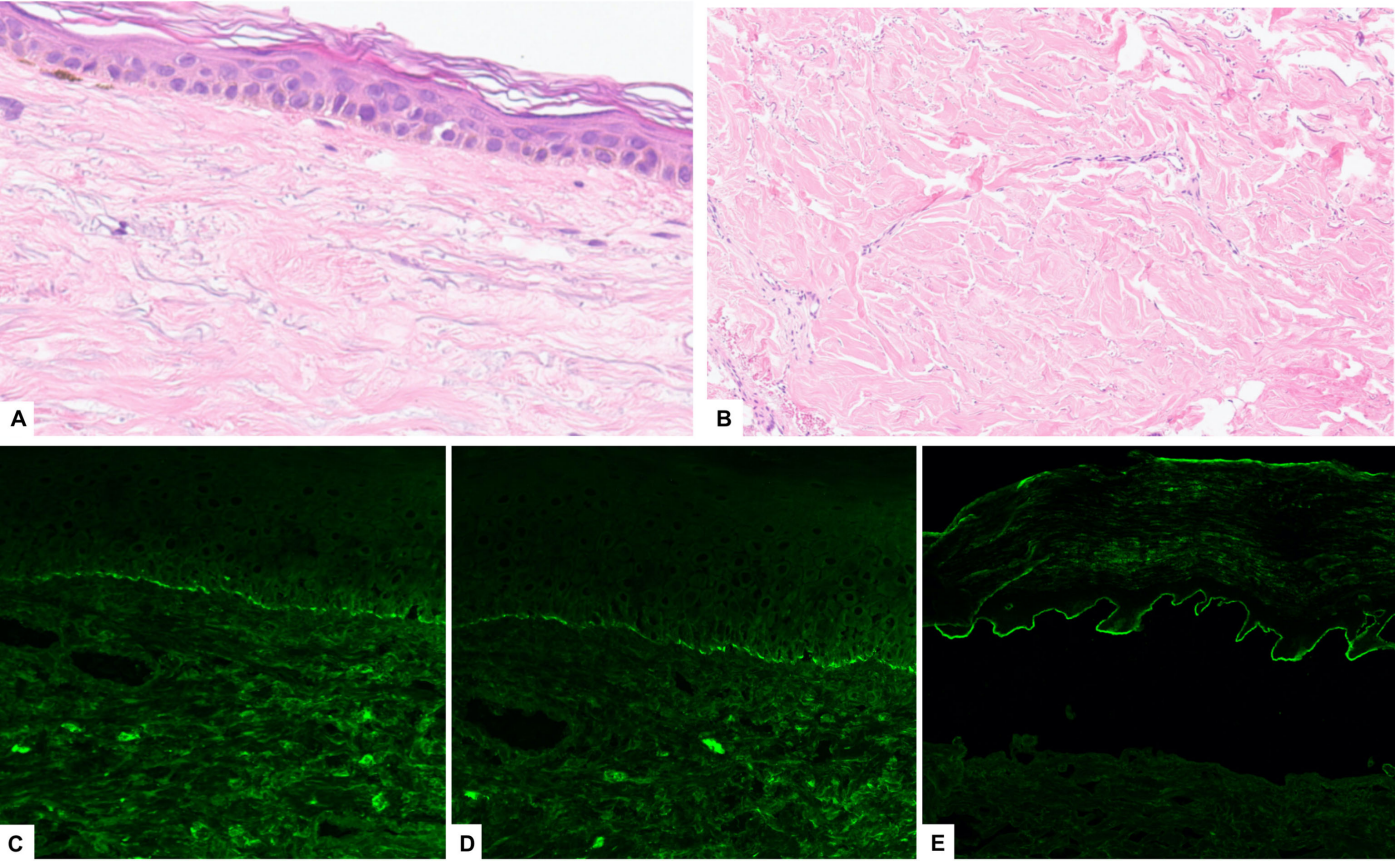
A skin biopsy from an indurated plaque of the trunk showing **(A)** epidermal atrophy and **(B)** thickened collagenous bundles in the reticular dermis (H&E). Direct immunofluorescence taken from the skin near to an erosion of the upper limb showed linear deposition of IgG **(C)** and C3 **(D)**, consistent with a diagnosis of BP; **(E)** indirect immunofluorescence of human salt-split-skin showing IgG deposition along the epidermal side of the basement membrane zone.

A biopsy obtained from one of the erosions of the upper left limb showed absence of the epidermis and a dermal inflammatory infiltrate composed of lymphocytes, histiocytes and rare eosinophil granulocytes. A skin biopsy was later obtained from an indurated plaque of the trunk, revealing findings consistent with LoS (**Figures 3A, B**).

Direct immunofluorescence (DIF) study from the perilesional skin at the left arm showed a linear deposition of IgG and C3 complement along the basement membrane zone (BMZ) (**Figures 3C, D**).

Indirect immunofluorescence (IIF) on salt-split-skin (SSS) showed a linear deposition of IgG autoantibodies along the epidermal-BMZ (**Figure 3E**). Enzyme linked immunosorbent assay (ELISA) showed elevated IgG antibodies to BP180 NC16A IgG (120 UI/mL; reference range below 9 UI/mL), but not to BP230. Collectively, the findings were consistent with a diagnosis of BP.

**Figure 4:**
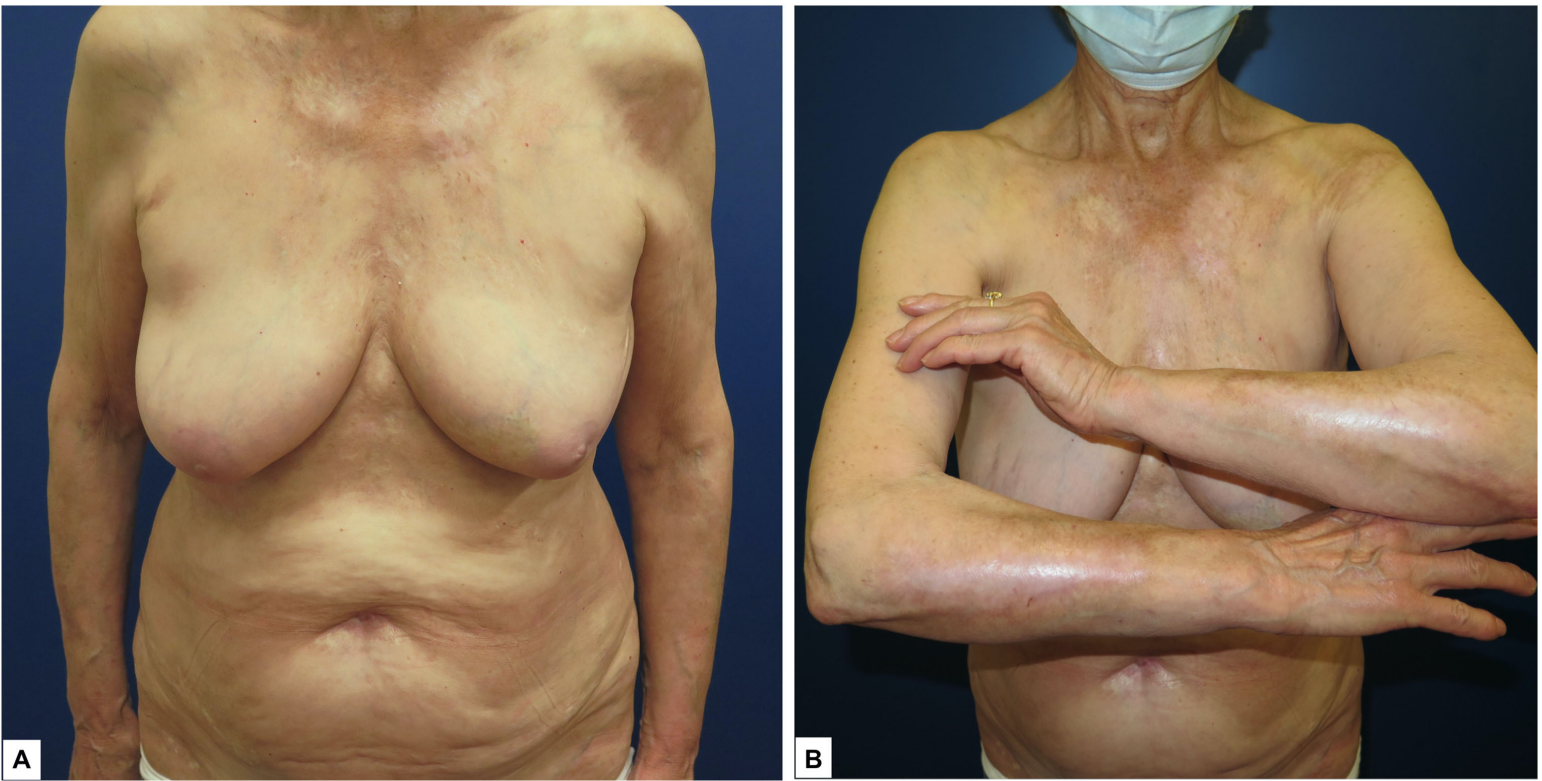
**(A, B)** Significant improvement of the lesions after the introduction of systemic steroids with complete resolution of the erosions after 4 months of treatment.

She was treated with a tapering course of oral prednisone starting from 0.5 mg/kg/day; topical clobetasol ointment twice daily was also added. After a 4-month follow-up, erosions completely healed, and pruritus disappeared (**Figures 4A, B**).

Serum samples were collected from the patient at 4, 8 and 12 months after treatment, while she was on clinical remission. SSS-IIF tested negative in all the serum samples. Anti-BP180 IgG antibodies were also below the cut-off value.

## Discussion

From the immunological and clinical point of view, this case can be regarded as a rare association between an autoantibody-mediated skin disease, such as BP, and sclerosing disorders, such as LoS and LiS. Interestingly, BP lesions were localized to area previously affected by LoS and LiS, without further extension. The patient’s serum demonstrated IgG antibodies exclusively to BP180 NC16A during active BP; these antibodies decreased clearly with steroid treatment and remained below the cut-off value during remission.

The coexistence between LoS, including plaque-type and generalized LoS, and LiS is well known, occurring from 5.8% to 38% of patients with LoS (20, 21). Conversely, we found only two cases of genital LiS (22, 23) and 3 cases of LoS who subsequently developed BP (24–26), including one case following phototherapy (24). Although our patient was previously treated with phototherapy, the long latency time makes unlikely a causal relationship between phototherapy and BP. The development of BP in patients with combined LoS and LiS is to our knowledge previously unreported.

Although they belong to different patterns of cutaneous inflammation (27), there are immunological overlaps possibly explaining the association of LoS and LiS with BP. As an example, T helper (Th) 2-derived cytokines, including interleukin (IL)-4 and IL-13, are implicated in sclerotic disorders such as LoS (28–30) and are also over-expressed in the skin and blood of patients with BP (4, 31); gene polymorphisms of IL-13 have been linked to an increased risk of BP as well as systemic sclerosis (28, 32). Antibodies to BP180 may be increased in patients with vulvar LiS (33–35), although they do not correlate with clinical activity and pruritus (36); moreover, in up to 40% of vulval LS patients, the NC16A domain of BP180 is a target for circulating T cells (37), a phenomenon that has been also reported in patients with lichen planus (38). Finally, the finding of the pemphigoid-predisposing human leukocyte antigen (HLA) haplotype, HLA DQ7, in patients with combined LoS and LiS (39, 40), supports a common genetic background between these diseases, explaining the frequent autoreactivity to BP180 in patients with LiS.

## Conclusion

To conclude, we reported a rare case of BP developed in a patient with pre-existing LoS and LiS. We hypothesized that LiS and LoS served as predisposing factors to the development of BP in our patient owing to the frequent T-cell reactivity to BP180 NC16A associated with LiS and the increased Th2-type signaling associated with LoS. An unknown external trigger had possibly induced a transient immunological shift precipitating autoantibody production and BP development. The main limitation of this study is that we were not able to collect peripheral blood samples before the emergence of BP. In fact, it would have been intriguing to analyze T-cell activation and serum autoantibody levels against BP180 during the pre-BP clinical stage. Further, HLA was not tested in our patient.

Finally, along with other experimental and clinical reports in the literature (41), this case suggests that different disease phenotypes, such as sclerosing dermatitis and BP, might be associated with an immune activation against the same autoantigen.

## Data Availability Statement

The original contributions presented in the study are included in the article/supplementary material. Further inquiries can be directed to the corresponding author.

## Ethics Statement

Ethical review and approval were not required for the study on human participants in accordance with the local legislation and institutional requirements. The patients/participants provided their written informed consent to participate in this study. Written informed consent was obtained from the individual(s) for the publication of any potentially identifiable images or data included in this article.

## Author Contributions

All authors were involved in drafting the article or revising it critically for important intellectual content, and all authors approved the final version. RM, EA, MB, and SG had full access to all data in the study and took responsibility for the integrity of the data and the accuracy of the data analysis. RM and EA conceived and designed the project. VM collected histopathological images. CP, FM, MCa, and MCe contributed to manuscript preparation and collection of clinical images.

## Conflict of Interest

The authors declare that the research was conducted in the absence of any commercial or financial relationships that could be construed as a potential conflict of interest.

## Publisher’s Note

All claims expressed in this article are solely those of the authors and do not necessarily represent those of their affiliated organizations, or those of the publisher, the editors and the reviewers. Any product that may be evaluated in this article, or claim that may be made by its manufacturer, is not guaranteed or endorsed by the publisher.
